# Mesenchymal Stem/Stromal Cell-Derived Small Extracellular Vesicles (MSC-sEVs): A Promising Treatment Modality for Diabetic Foot Ulcer

**DOI:** 10.3390/bioengineering10101140

**Published:** 2023-09-28

**Authors:** Dimitrios Kouroupis, Lee D. Kaplan, Camillo Ricordi, Thomas M. Best

**Affiliations:** 1 Department of Orthopaedics, UHealth Sports Medicine Institute, Miller School of Medicine, University of Miami, Miami, FL 33146, USA; kaplan@med.miami.edu (L.D.K.); txb440@med.miami.edu (T.M.B.); 2 Diabetes Research Institute & Cell Transplant Center, Miller School of Medicine, University of Miami, Miami, FL 33136, USA; cricordi@med.miami.edu

Diabetic foot ulcer (DFU) is associated with neuropathy and/or peripheral artery disease of the lower limb in diabetic patients, affecting quality of life and leading to repeated hospitalizations and infections. Importantly, approximately 26 million people worldwide have a DFU annually, with around 20% suffering from moderate and severe DFU resulting in amputation [1]. The multifactorial pathology of DFU makes its treatment challenging. Standard of care includes debridement, infection control, optimization of blood flow, and offloading; however, with these treatments, only 50% of patients heal within 20 weeks and 50% recur within 18 months [2]. Therefore, there is an urgent need for novel and effective therapeutic modalities.

In situ, factors that result in slow or nonhealing DFUs are the decreased number of infiltrating macrophages, abnormal concentration of pro-inflammatory cytokines, reduced concentration of reparative growth factors, reduced collagen synthesis, and, most importantly, the deranged neovascularization. Therefore, novel therapeutic approaches should target three main mechanisms to resolve DFU, namely immunomodulation, neovascularization, and matrix formation. On this basis, mesenchymal stem/stromal cell (MSC)-based therapy has gained attention given its immunomodulatory, anti-inflammatory, anti-fibrotic, pro-angiogenic properties [3,4,5], and effects on monocyte/macrophage phenotypic polarization [6]. These collective effects this therapy an attractive candidate to simultaneously alter various stages of DFU healing. Specifically, MSC can contribute to DFU resolution by targeting both cellular and molecular mechanisms (reviewed in [7]). At the cellular level, MSC induces fibroblast proliferation, ECM production, and endothelium/epithelium stabilization. Also, MSC attenuates local inflammation by polarizing M1 pro-inflammatory macrophages to M2 alternative phenotype and by increasing the production of regulatory T cells. Importantly, at the molecular level, MSC decreases the secretion of pro-inflammatory cytokines such as IL-1, IL-6, TNF-α, and induces the secretion of anti-inflammatory molecules such as IL-4, IL-10, and TGF-β. Finally, MSC suppresses pain by specifically modulating local neurogenic inflammatory/immune responses. For example, substance P (SP), a neurotransmitter and a modulator of pain perception, is secreted locally and increases vascular permeability, favoring immune cell infiltration, while directly affecting macrophage phenotypic polarization and migration to sites of inflammation. Based on our findings, SP can be degraded by MSC highly expressing cell-membrane-bound (ectoenzyme) neutral endopeptidase CD10/neprilysin, resulting in a reduction in inflammation and analgesia [8]. From 2008 to date, clinical trials have been performed that mainly administer MSC via intramuscular and intradermal routes, with significant therapeutic outcomes regarding wound healing and recurrence rate.

In parallel, as part of a continuous effort to unravel the MSC-based therapy underlying mechanisms of action, the appearance of small extracellular vesicle (MSC-sEV)-enriched fractions within their secretome has started to contribute to that elucidation. MSC-sEVs are nanosized (50–200 nm) vesicles generated via the endosomal pathway and secreted by numerous cells in response to their surrounding milieu. Therefore, their cargo (i.e., proteins, miRNAs, lncRNAs, circRNAs) and lipid shell may carry information that reflects particular changes in the parental cells’ microenvironment, specifying intrinsic communications to proximal or distal sites. From a clinical safety standpoint, xeno-free, regulatory-compliant formulations for parental MSC processing, such as the human pooled platelet lysate, can result in purified and hypo-immunogenic (lack of MHC-II and low expression of MHC-I) MSC-sEVs. Specifically, MSC-sEVs can modulate the sequential stages of DFU healing by regulating the functionality of cells in the in vivo niche involved in the attenuation of excessive inflammation, ECM production and neovascularization. Upon delivery of MSC-sEVs cargo, they activate the PTEN/Akt signaling pathway to polarize macrophages, activate Erk1/2 or eNOS/AKT/ERK/P-38 and inhibit AP-1/ROS/NLRP3/ASC/Caspase-1/IL-1β signaling pathways to promote endothelial cell function recovery and angiogenesis, and activate PI3K/Akt or Rho-YAP signaling pathways to induce fibroblasts’ proliferation and collagen production (reviewed in [9]). Interestingly, recent studies demonstrated the MSC-sEVs’ senotherapeutic effects that induce the apoptosis of pro-inflammatory senescent cells and mitigate the adverse effects of senescence-associated secretory phenotype signaling in vivo [10]. For enhanced therapeutic outcomes, we and others have demonstrated the ability to customize MSC-sEVs contents and identity (e.g., CD10^High^ or CD146^High^) by processing in vitro parental MSC with specific protocols tailored to a specific therapeutic function (e.g., CD10-dependent SP degradation), which is designed to reduce inflammation/pain [11,12,13]. Customized MSC-sEVs contain therapeutic miRNAs and proteins that are directly involved in macrophage polarization, T cell activation, and the regulation of inflammatory cytokine transcription, as well as pro-angiogenic/reparative actions in vivo. Therefore, a standardized “off-the-shelf” product with high reproducibility and low variability for allogeneic therapeutic schemes can evolve. Additionally, biomaterials can be used as carriers for MSC-sEVs. Preclinical studies have showed that MSC-sEVs, combined with hydrogels and other specialized biomaterials, control their release at the wound site and promote wound angiogenesis, wound granulation tissue formation, re-epithelialization, and collagen remodeling (reviewed in [14]).

Overall, up-to-date research findings suggest that MSC-sEVs are a promising therapeutic modality for DFU. Specifically, MSC-sEVs can attenuate inflammation, promote angiogenesis, and improve ECM formation. On this basis, the development of innovative cell-free, sEV-based therapeutic approaches and the incorporation in their manufacturing of regulatory-compliant practices can facilitate the translation of proof-of-concept preclinical data into effective clinical protocols.
